# In-reach specialist nursing teams for residential care homes: uptake of services, impact on care provision and cost-effectiveness

**DOI:** 10.1186/1472-6963-8-269

**Published:** 2008-12-22

**Authors:** Ala Szczepura, Sara Nelson, Deidre Wild

**Affiliations:** 1 Warwick Medical School, University of Warwick, Coventry, West Midlands, UK; 2 Faculty of Health and Social Care, University of the West of England, Bristol, UK

## Abstract

**Background:**

A joint NHS-Local Authority initiative in England designed to provide a dedicated nursing and physiotherapy in-reach team (IRT) to four residential care homes has been evaluated. The IRT supported 131 residents and maintained 15 'virtual' beds for specialist nursing in these care homes.

**Methods:**

Data captured prospectively (July 2005 to June 2007) included: numbers of referrals; reason for referral; outcome (e.g. admission to IRT bed, short-term IRT support); length of stay in IRT; prevented hospital admissions; early hospital discharges; avoided nursing home transfers; and detection of unrecognised illnesses. An economic analysis was undertaken.

**Results:**

733 referrals were made during the 2 years (range 0.5 to 13.0 per resident per annum) resulting in a total of 6,528 visits. Two thirds of referrals aimed at maintaining the resident's independence in the care home. According to expert panel assessment, 197 hospital admissions were averted over the period; 20 early discharges facilitated; and 28 resident transfers to a nursing home prevented. Detection of previously unrecognised illnesses accounted for a high number of visits.

Investment in IRT equalled £44.38 per resident per week. Savings through reduced hospital admissions, early discharges, delayed transfers to nursing homes, and identification of previously unrecognised illnesses are conservatively estimated to produce a final reduction in care cost of £6.33 per resident per week. A sensitivity analysis indicates this figure might range from a weekly overall saving of £36.90 per resident to a 'worst case' estimate of £2.70 extra expenditure per resident per week.

Evaluation early in implementation may underestimate some cost-saving activities and greater savings may emerge over a longer time period. Similarly, IRT costs may reduce over time due to the potential for refinement of team without major loss in effectiveness.

**Conclusion:**

Introduction of a specialist nursing in-reach team for residential homes is at least cost neutral and, in all probability, cost saving. Further benefits include development of new skills in the care home workforce and enhanced quality of care. Residents are enabled to stay in familiar surroundings rather than unnecessarily spending time in hospital or being transferred to a higher dependency nursing home setting.

## Background

The number of older people aged 65 and over in the UK is predicted to rise significantly in the next 20 years, with the number of advanced age (85 and over) expected to increase by two-thirds [1-5]. As many illnesses increase with advancing age and lead to disability, this will give rise to increased continuing health and social care needs. In 2002, 900,000 older people in the UK had a high level of need i.e. were unable to carry out one or more activities of daily living. Over the coming two decades this number is predicted to increase by 54 per cent. With the increase in numbers of frail elderly people, social care costs are likely to grow rapidly, potentially more quickly than health care costs [5].

At the same time, changes in patterns of health care provision have resulted in fewer UK hospital beds, a reduced length of stay and increased reliance on community services [6]. The number of hospital beds for older people has fallen by nearly half, focusing the NHS role on acute care provision for this population. Over time, the residential care home sector has become an increasingly important source of long-term care provision for older people. As the number of people with impairment and dependency grows over the coming years, this will increase the pressure on social and health care services. The issue of future care for older people and how this can best be provided and funded is therefore of increasing importance [5,7-10].

Currently, 1.2 million people aged 65 and over use publicly funded social care services. Local authorities spend £8 billion on personal social care services, with almost 60 per cent of this expenditure for placements in residential and nursing homes [5]. Spending on care home placements has risen more rapidly than on home care, and is predicted to continue to do so [5].

There are currently approximately 19,000 residential and nursing homes for adults in England with a total capacity of 441,000 places. Regulatory and other pressures on the sector have led to a fall in the number of places in the last few years, with 20,000 lost in the period 2003–2005 [11]. Residential homes provide personal and social care for people who are no longer able to live in their own home. Nursing and medical care is usually provided through general practitioners (GPs) and district nurses (DNs). Nursing homes provide nursing care in-house as well as personal and social care, with qualified nurses employed in the home to provide the nursing care. Most care homes are small providers; the average home for residents aged 65 years plus has 35 beds. At the same time, the sector is becoming increasingly concentrated with fewer, larger care homes [12].

Both health and social care policy makers accept that there is considerable potential to reduce unplanned admissions to hospital and unnecessary moves into higher dependency care for older people. A number of Partnership for Older People pilots (established in 2006/7 and 2007/8) are striving to develop innovative approaches to joint working aimed at improving outcomes and reducing the use of unscheduled inpatient care by older people [13]. The potential to replace acute bed days with less intensive beds is considerable as explained in the White Paper *Our Health, Our Care, Our Say *[13].

An important factor limiting the role of residential care homes in these developments is their limited access to nursing skills. The issue of nursing care provision in care homes is complex. In October 2001, the government extended the provision of NHS-funded nursing care in England to residents in nursing homes [14]. The NHS contribution towards expenditure on nursing care in nursing homes was around £550 million in 2005/6. Residential homes, however, are excluded and are reliant on support from district nurses which has been identified as largely *ad hoc *[15]. It has been suggested that if residential care home staff were more skilled in anticipating health problems in residents or in delivering care district nurse input could be reduced and staff roles enhanced [16].

The challenge of providing quality, long-term clinical care in residential homes is not unique to the UK. Policy makers, clinicians and care home staff in many countries are increasingly aware of the importance of facing up to this challenge for older people. This paper describes a study which has evaluated the impact of a new model of nursing care provision for residential care homes.

## Methods

### Setting

The study was carried out in Bath and North East Somerset, England. The Local Authority (LA) and Primary Care Trust (PCT) provide care for a population of 169,040 residents; of these, 30,160 (18%) are 65 and over and 8,400 (4.9%) are aged 80 plus [17]. 1,017 individuals were living in a care home at the time of the 2001 census; 570 in a nursing home and 447 in a residential home.

In 2005, a new service was introduced by the PCT and LA for a group of local authority residential care homes caring for 131 long-term residents in four homes. This provides 24-hour cover seven days per week via a specialist in-reach team (IRT) which offers dedicated nursing and physiotherapy input. The team also offers *in situ *support for a maximum of 15 'virtual' beds at any one time for specialist nursing in the care homes e.g. to provide on-site care to prevent transfer to hospital or to higher dependency care in a nursing home. In addition, the IRT team provides support for up-skilling designated care home staff through enhanced health training to NVQ3 level. The project was awarded Bath & North East Somerset Research Ethics Committee approval (ref: 05/Q2001/247) on 17th November 2005.

Table 1 shows the characteristics of the study care homes and their residents.

**Table 1:** Characteristics of Residents and Care Homes

**Descriptor**	**Characteristics of Study Homes**
*Resident demographics*:	
Total number of residents	131 (100%)
Male residents	37 (28%)
Female residents	94 (72%)
Resident age: Mean (range)	87 (71–104 years)
Male resident age: Mean (range)	87 (71–98 years)
Female resident age: Mean (range)	87 (74–104 years)
*Resident dependency levels*:	
RNCC banding^1^: Low: medium: high	56% (L); 44% (M); 0% (H)
Mean Barthel score^2 ^(range)	71.4 (18–95)
*Care home characteristics*	
Number of IRT beds	15
Number of care home staff^3^	70
Number of staff designated as IRT new role carer support staff (NVQ3 level)	20

^1^'RNCC. *Medium banding *= multiple care needs; will require intervention of registered nurse on at least a daily basis; may need access to a nurse at any time; condition stable and predictable, and likely to remain so if treatment and care regimes continue. *Low banding *= care needs can be met with minimal RN input in setting such as residential care home, with support from district nurse.

^2 ^Barthel: Scores range from 0 to 100. Higher score = more independent.

^3 ^At beginning of project 2006, excludes managers and domestic staff.

The nursing needs of a sample of 1 in 4 residents were measured midway through the study (May 2006); the sample excluded dementia cases. The Minimum Data Set (MDS) care assessment tool was used to classify these 36 residents into Registered Nurse Contribution to Care (RNCC) bandings based on an algorithm [14]. A modified Barthel score (excluding mental health and pain) was also used to record residents' ability to carry out Activities of Daily Living in the same sample of 36 residents, as shown in Table 1.

### Data collection and analysis

Key measures indicative of service delivery and quality were identified based on a review of the literature and interviews with key stakeholders. These measures included: the number of residents referred to IRT; reason(s) for referral; outcome of IRT triage (e.g. admission to IRT bed, short-term IRT support); length of stay in IRT; type of IRT intervention (if any); number of referrals to hospital, with clinical condition; number of early hospital discharges with type of follow-up service; and detection of hitherto unknown illnesses with conditions identified. Admission to the IRT service and prioritising of individuals for clinical management were guided by a clinical risk stratification tool developed for this purpose (see Additional file 1). Structured proformas were used to capture this data prospectively over the period July 2005 to June 2007. Information was entered by IRT and care home staff at the time of referral and during the care episode; completed forms were collected monthly by the field researcher.

Classification of the reason for each referral to IRT was recorded as part of the referral process. The clinical condition was agreed after scrutinising a resident's notes. All referrals were coded with a primary diagnostic code where possible and any relevant secondary diagnoses. For referrals with no clear clinical diagnosis, cases were assigned retrospectively to a series of non-clinical categories developed by a review panel (see below).

Certain measures required professional judgement e.g. a prevented hospital admission or an avoided transfer to a nursing home. All such cases were assessed retrospectively by a review panel made up of external as well as internal members. All referrals to IRT which might have prevented a hospital admission were examined by the review panel in combination with the clinical diagnosis, data from the clinical risk stratification tool, and other information from the resident's notes. Four final categories were used: 'Yes' (hospital admission prevented); 'Yes probable'; 'Improbable'; and 'No' (see Additional file 1 for definitions). This allowed for necessary levels of certainty in prevention of an event might or might not have occurred. A similar approach was adopted for identifying averted transfers to a nursing home. Completed forms were collated by the IRT administrator for computer entry. Quality checks were undertaken by the research team for all data items entered.

The cost of the new service was estimated once it had stabilised. Costs were calculated to include: nursing, physiotherapy and administrator wages/salaries; salary oncosts; travel; consumables; and capital (accommodation and equipment). Savings were quantified linked to: avoided hospital admissions; reduced hospital stay through early discharge to IRT care; and prevention of transfers to a more expensive nursing home setting. Other longer term savings resulting from improved preventive care (e.g. earlier detection of particular conditions and prompt treatment) were also estimated. Costs, cost savings and additional non-financial benefits for residents were compared.

## Results

### Referral patterns

A total of 733 referrals were made over the 2 year period, with an annual mean number of 5.6 per resident (range 0.5 to 13.0). Referrals resulted in a total of 6,528 visits by members of the in-reach team (mean visits per referral = 8.9). The average number of IRT visits per month to the group of care homes was 272. An audit of district nurses visits to the care homes in a one month period recorded only 28 visits. Thus, the new service appears to have identified a significantly higher level of nursing need, over and above that identified by existing *ad hoc *district nursing services.

Table 2 indicates the main reason for referral to the IRT. Two thirds of referrals were aimed at maintaining the resident's independence in the care home. Just over one quarter (28%) had the objective of preventing an A&E attendance or an unplanned hospital inpatient admission. A much smaller number (3%) were to facilitate an early discharge from hospital; and 2% were specifically aimed at preventing transfer to a nursing home.

**Table 2:** Reasons for Referral to In-Reach Team (N = 733)

**Reason for Referral**	**Frequency** **N (%)**
To maintain independence in residential home	486 (66)
To prevent hospital admission	198 (27)
Prevent A&E attendance	8 (1)
Prevent admission to nursing home	17 (2)
Facilitate early/safe discharge	20 (3)
Routine observations	4 (1)

**Total**	**733 (100)**

**Figure 1:**
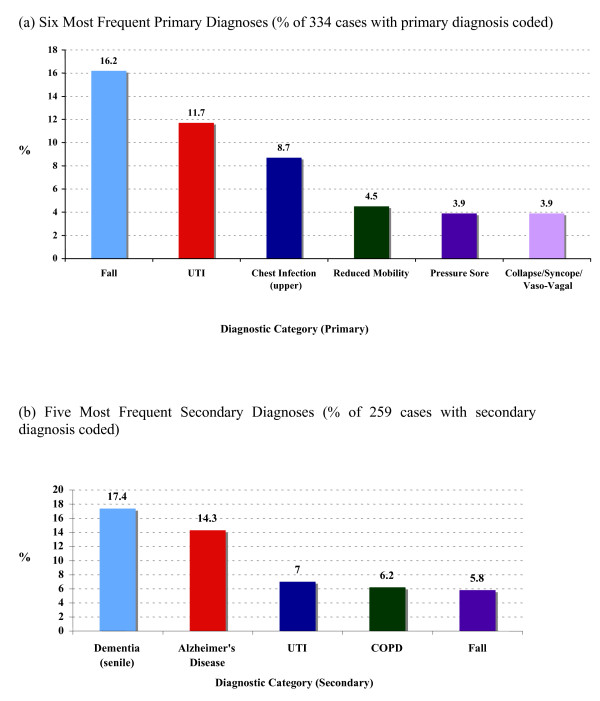
**Main Primary and Secondary Diagnoses for In-Reach Team Referrals**. (a) Six Most Frequent Primary Diagnoses (% of 334 cases with primary diagnosis coded). (b) Five Most Frequent Secondary Diagnoses (% of 259 cases with secondary diagnosis coded).

46% (334/733) of referrals had an identifiable primary diagnosis, and one in three (259/733) a secondary diagnosis. Figures 1a and 1b show the five most common primary and secondary clinical diagnoses respectively. Falls and infections (chest and UTI) constituted the vast majority (83%) of primary diagnoses. Dementia and Alzheimer's disease made up over half of identified secondary diagnoses.

The remaining 399 referrals with no primary clinical diagnosis fell into non-clinical categories as shown in Table 3. Slightly over half were classified as 'opportunistic partnership' activities i.e. referrals to IRT to deal with something which would normally be undertaken by a district or practice nurse. The second largest group, just over one quarter, fell into the category of 'advice only' or 'telephone triage'. A common example in this category related to medication for pain or indigestion prescribed on an 'as required' basis.

**Table 3:** Other General Descriptions of In-Reach Team Referrals (N = 399)

**Other Reasons for Referrals**	**Frequency** **N (%)**
Opportunistic partnership (DNs/Practice nurse)	217 (54%)
Advice only (includes telephone triage)	102 (26%)
Incomplete paperwork	27 (7%)
Physiotherapy only (advice and assessment)	20 (5%)
Inappropriate referral	22 (6%)
Other^1^	11 (3%)

**Total**	**399 (100%)**

^1^Includes: Care home staff unable to facilitate discharge (n = 3); Care home staff reluctant for IRT involvement (n = 2); Uncertain diagnosis (n = 2); Single other (n-4)

The level of inappropriate referrals, i.e. those judged not to require the input of a specialist nursing team member, was low (6%).

Table 1 shows that the results of assessments carried out using MDS indicate nursing needs in a majority of residents, whereas care staff's routine Barthel scores, based on residents' ability to carry out Activities of Daily Living, indicated dependency needs in only a minority of residents drawn from the same population. As the two scores measure different things, this should not be viewed as conflicting evidence. For example, a resident with dementia can be functionally independent yet have major, often un-communicated health/nursing needs.

### Activities undertaken by in-reach team

Activities carried out by the IRT during care home visits, supported by care home staff with enhanced health training to NVQ3, are shown in Table 4. Over one third were classified as general nursing care; assessment and observation represented a further 12%. The remaining activities were wide ranging, although most focused on aspects of clinical care.

**Table 4:** In-Reach Team Activity During Care Home Visits (N = 6,528)

**Type of Activity**	**Frequency** **N (%)**
General nursing care	2,372 (36)
Assessment	431 (7)
Basic Observations	292 (5)
Nursing Intervention	279 (4)
Diet and Fluid Intake	249 (4)
Pressure area care	212 (3)
GP liaison	203 (3)
Discharge visit	177 (3)
Medication	172 (3)
Terminal Care	166 (2)
Catheter Care	154 (2)
Support Worker training	105 (2)
Other^1^	1,611 (29)

**Total**	**6,528 (100)**

^1 ^Recorded fewer than 100 times. Include: basic monitoring, bowel care, continence care, skin care, non-surgical wound care, and liaising with other health care professionals.

Table 5 shows that the vast majority (82%) of referrals did not require admission to an IRT bed and were triaged to short-term IRT support. This support was limited to a maximum of 3 patient contact episodes. After 3 contacts, residents were assessed again as to their appropriateness for admission to longer-term IRT bed care. A clinical risk tool was used to assess each resident's level of risk e.g. for hospital admission, and their need for IRT services or for referral to external community health professionals.

**Table 5:** Outcome of Referrals to In-Reach Team (N = 733)

**Type of Outcome**	**Frequency** **N (%)**
Short-term IRT support only^1^	602 (82)
Accepted into IRT bed service	118 (16)
Inappropriate referral	5 (<1)
GP call out	4 (<1)
Emergency services call out	2 (<1)
Not recorded	2 (<1)

**Total**	**733 (100)**

^1^Includes some cases with more than 3 contacts (originally set as limit) – these might be categorised as 'IRT monitoring' rather than short-term support i.e. periodic assessment & care co-ordination.

**Figure 2:**
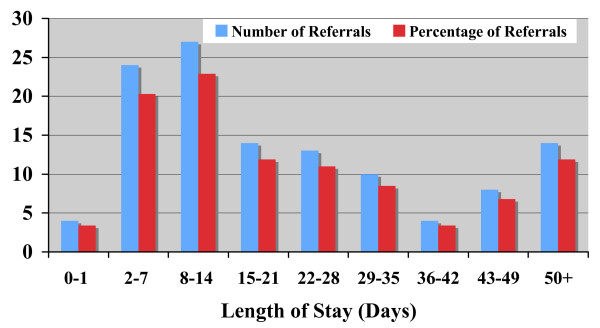
**Length of Stay in In-Reach Team Beds**.

The 118 admissions to an IRT supported bed represented 70 residents, thus some residents had more than one admission. The most common reasons for admission to an IRT bed were falls (11%), chest infection (9%); urinary tract infection (9%); or reduced mobility (9%). There was also a small number of palliative care referrals (7/118) managed through IRT admission. Other conditions dealt with by admission to an IRT bed included: angina, carcinoma, chronic congestive cardiac failure, cerebral infarction, pneumonia, anxiety and abnormal weight loss. The average length of stay was 25 days (range 1 to 125). Figure 2 shows a bimodal distribution with a small number of residents staying longer than 50 days. In total, the 15 IRT beds were occupied for 2,949 days, with a maximum of 18 beds occupied at any particular time (i.e. 120% occupancy). Analysis indicates that the total number of bed days rose from 1,121 days in 2005/06 to 1,581 in 2006/07 (41% increase) as the service became established. Cases also became more complex with median stay rising from 12.0 to 25.5 days.

**Figure 3:**
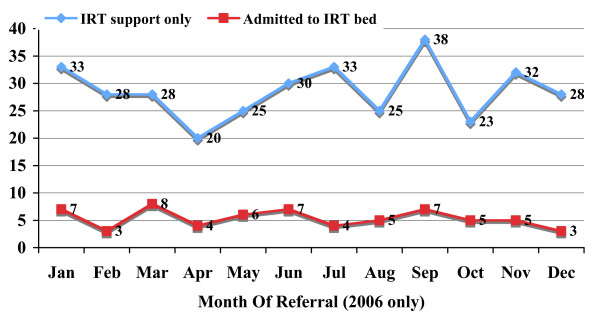
**Seasonal Variation in Outcomes of Referral to In-Reach Team**.

Figure 3 confirms that there was no clear seasonal pattern in long-term vs. short-term support for referrals to the in-reach team. Of the 602 episodes triaged to short-term support, 213 could be linked to a clinical diagnosis; the remaining 389 were classified as opportunistic partnerships etc. Referrals with a diagnosis triaged to short-term care were mostly for falls (19%) and urinary tract (14%) or chest (12%) infections. Other conditions dealt with by the IRT team without admission to an IRT bed included diabetes, constipation, vomiting, head or other injury, joint pain and chronic obstructive pulmonary disease (COPD).

### Hospital admissions avoided

Table 6 shows the panel's judgement on whether hospital admission was avoided for residents admitted to an IRT bed. In 61% of cases, IRT admission was judged to have certainly or probably prevented a hospital admission. In these cases, the review panel also assessed whether a short (less than 48 hours) or longer hospital stay would have been expected. For the vast majority (96%) of cases for which this could be assessed, length of stay (LOS) was predicted to be longer than 48 hours.

**Table 6:** Prevented Hospital Admissions-Residents Admitted to IRT Bed (N = 118)

**Hospital Admission Prevented?**	**Frequency** **N (%)**
Yes	39 (33)
Yes, Probable	33 (28)
Improbable	18 (15)
No	10 (9)
Not assessable	18 (15)

**Total**	**118 (100)**

Table 7 shows the level of admissions prevented for all referrals to IRT with a primary diagnosis; 34% were judged to have certainly or probably prevented a hospital admission. This is lower than the level for residents admitted to an IRT bed, as might be expected (see Table 6). For the remaining referrals with no diagnostic code, it was difficult to decide whether an admission had been avoided. If a frequency half that estimated in cases with a recorded diagnosis is assumed, a total of 197 prevented admissions would be identified.

**Table 7:** Prevented Hospital Admissions-All Referrals to IRT (N = 335)

**Hospital Admission Prevented?**	**Frequency** **N (%)**
Yes	47 (14)
Yes, Probable	68 (20)
Improbable	82 (24)
No	30 (9)
Not assessable	108 (32)

**Total**	**335 (99)**

It is possible to estimate the likely cost saving associated with the admissions prevented over 2 years in two main ways. Firstly, using the average cost of a hospital admission (£2,000), this would suggest a total saving of £394,000 for 197 prevented admissions over the two years. Secondly, under the charging regime for the PCT, an inpatient episode costs £800 for a stay less than 48 hours and £2,500 for a longer stay. Since 96% of avoided admissions were judged to require an inpatient stay of over 48 hours, this approach would indicate a higher saving of £479,104 for the same number of prevented admissions over two years. If the frequency of avoided admissions in cases with no diagnostic code differed from that assumed above (for example, ranging from 20% to 80% of that observed in cases with a diagnosis recorded) then the estimated cost saving would range from £284,000 to £539,904. In the first instance, a conservative figure of £406,000 saving over two years was assumed. If the saving in emergency ambulance call out costs (£48,462 for these avoided admissions at £246 per call [18]) is added, this produces an estimated saving of £227,230 per annum.

### Early hospital discharges facilitated

Access to the IRT service was judged to have facilitated 20 early discharges from hospital over the observation period. Approximately two thirds (13/20) were discharged to an IRT bed; one was triaged to short-term IRT support; 4 were judged suitable to hand over to community nursing services; and a further 2 were capable of a direct return to the home without community nursing support but with advice to the home manager to re-contact IRT if any problems arose. The majority (12/20) of referrals to IRT were made by the care home managers, following contact from the hospital discharge service. In the remaining 8/20 of cases, direct contact was made with IRT by the hospital staff.

The average length of stay for the 13 early discharges admitted to an IRT bed was 20.3 days (range 2 to 78 days); the total number of days was 264. Assuming the same length of hospital stay has been avoided, and that the other 7 early discharges each saved only one tenth of this figure, a total saving of £69,553 over two years is estimated, or £34,777 per annum.

### Nursing home transfers prevented

31% (37/118) of admissions to an IRT bed were judged to have helped prevent transfer to a nursing home; 25/118 with a high level of certainty. Because, even if transfer is delayed, it may not necessarily be avoided entirely in the longer term, cost savings are difficult to estimate. Also, a number of episodes may relate to the same resident. Further analysis identified 28 individual residents for whom transfer was prevented. The delay recorded during the study period ranged from 2 to 23 months for individual residents (average 11.9 months up to June 2007). For many it appeared that transfer had been prevented beyond this date and for the foreseeable future.

Preventing a move from residential care to an independent nursing home will decrease LA expenditure by £18,011 for each 12 months delay for these 28 residents. Once transferred to a nursing home, an NHS contribution for nursing care will also be required [14]. The level of payment will depend on the RNCC banding group into which an individual falls. Table 1 indicates that 56% of a cross-section of residents fell into the low equivalence band and the remainder into the medium band. Assuming similar nursing needs in those at risk of transfer, there would be a saving to the PCT of £85,787 (2006 RNCC rates) for every 12 months delay.

Prevented transfers are thus estimated to produce an overall saving of £103,798 over two years for the PCT and LA based on an average one year's delay, or £51,899 per annum. This figure will be higher if, as seem likely, the residents at risk of transfer are in higher RNCC bandings than the cross-section of residents assessed, or if delayed transfer is longer than 12 months.

### GP and community nurse visits avoided

Overall, 80/118 (68%) of admissions to an IRT bed (average LOS 25 days) were judged to have prevented one or more GP visits. If one GP visit is avoided each week this will lead to an estimated cost saving of £19,734 over 2 years, based on the reported cost of a GP home visit including travel time [18]. Similarly, for the 217 community nurse visits avoided through IRT dealing with something which would normally require these staff (see Table 3), an estimated further saving of £4,991 is predicted over two years [18]. In total, a saving of £12,363 per annum is estimated through prevention of these visits by IRT.

### Previously undetected illnesses

A further important benefit provided by IRT working proactively with care home staff was the identification of previously undetected illnesses or conditions. The review panel found that in 57% (192/334) of cases with a clinical diagnosis recorded which were referred to IRT, a previously unrecognised illness was detected (see Table 8). The three most common conditions were UTI, chest infections and constipation. There was evidence of a small year-on-year increase in the number of illnesses identified from 2005/06 to 2006/07.

**Table 8:** Previously Undetected Illness Cases by Clinical Diagnosis (N = 192)

**Undetected Illness/Condition**	**Frequency** **N (%)**
UTI	44 (23)
Chest Infection	26 (14)
Constipation	22 (12)
Dehydration	9 (5)
Localised oedema	8 (4)
Pressure Sore	7 (4)
Hypotension	5 (3)
Other^1^	71 (37)

**Total**	**192 (100)**

^1 ^Other = Conditions with fewer than 5 cases. Include: abnormal weight loss, CVA, malnutrition, pneumonia, polypharmacy, cellulitis etc.

Estimating the likely cost savings associated with this type of early detection is difficult. Assuming a conservative figure (that one in every ten cases avoids a future hospital stay) this would represent a cost saving of £19,200 per annum. If the number of cases detected is higher (since this estimate is based only on the 46% of referrals with a clinical diagnosis recorded), the saving will be proportionately higher. Non-financial benefits, in terms of improved health and quality of life, will be especially important in cases of previously undetected illnesses.

These findings, combined with assessments carried out using MDS and routine Barthel scores (see Table 1), do suggest it is important for residents to receive a more comprehensive routine health assessment, than one which is focused on functional Activities of Daily Living, as a precursor for better care planning and intervention. This has implications both for the knowledge level required by care home staff taking on 'enhanced' roles and for the level of support they may require from a nurse.

### Cost of in-reach team service

The annual cost of the specialist in-reach team is shown in Table 9 based on actual expenditure once the intervention was stabilised (2005/06). The final column provides indicative costs for a 'Shared Care' model in which a core IRT nursing team works with existing community professionals, drawing on various specialist staff (e.g. physiotherapist, occupational therapist, and registered mental nurse) when required rather than including these in the core team. The introduction of a shared care model is expected to reduce costs from £302,000 to £253,000 per annum.

**Table 9:** Cost of In-reach Team and Shared Care Model

**Cost Item**	**Annual Expenditure** **(2005/06 prices)**	**'Shared Care' Model^1^** **(2007/08 prices)**
Salaries^2^: IRT Nursing staff	242,368^3^	218,000^4^
Salary Physiotherapist	6,601	-
Salaries IRT Administrator^5^	18,704	9,500
Travel/lease cars	12,335	12,000
Uniforms/clothing allowance	629	629
Accommodation & services e.g. electricity	5,000^6^	5,000
Office costs e.g. telephone, stationery etc	1,992	1,900
Office equipment e.g. PCs, photocopier etc	11,958	3,000
Clinical equipment & consumables	2,726	2,800

**TOTAL COST**	302,313	252,829

^1 ^Shared Care/Locality model, in-reach team draws on existing community staff (i.e. mental health nurses, physiotherapists, occupational therapists etc) on as and when required basis

^2 ^Salaries include overhead costs (such as employer's National Insurance Contributions and pensions). Expenditure excludes one nurse assessor funded for 12 months (Skills for Care)

^3 ^Core team (not fully established until November 2005) consisted of 5 WTE band 5 nurses, 3 WTE band 6 nurses & 1 WTE band 7 nurse. In addition, 18 hrs per week physiotherapist input.

^4 ^Core team for Shared Care/Locality Model consisting of: 4 WTE band 5 nurses (working 7 am – 9 pm seven days/week) & 1 WTE band 7 nurse. Team draws on other community staff (see 1 above).

^5 ^WTE administrator in 2005/6; 0.5 WTE administrator in Shared Care/Locality Model.

^6 ^Based on actual charge levied for accommodation in new Resource Centre (2007)

Table 10 provides a comparison of the cost of the IRT service with observed savings. The table shows that the largest savings are linked to avoided hospital admissions, followed by delayed transfers to nursing homes and early discharges from hospital. Displaced GP and community nurse visits save the smallest amount. Early detection of illness is difficult to quantify in monetary terms, but appears to offer a similar level of saving. The overall effect is cost saving with an overall estimated annual saving of £43,000 per annum or £6.33 per resident per week. In a 'Shared Care' model, this saving might rise to £92,600 per annum or £13.60 per resident per week.

**Table 10:** Annual Cost of In-reach Team and Estimated Savings

**ITEM**	***Annual Value*^1 ^*(£)***
***INTERVENTION COST***	
Annual total IRT cost^2^	+302,313
**Average cost/resident/week**	+44.38
	
***ESTIMATED SAVINGS***	
Avoided hospital admissions	-227,230
Early hospital discharges	-34,777
Delayed/prevented nursing home transfers	-51,899
GP & visits avoided	-12,363
Early detection of illness	-19,200
**Annual savings**	-345,469
	
***INCREMENTAL COST (EXPENDITURE - SAVINGS)***	
**Total annual incremental cost**	-43,156

**Average incremental cost/resident/week**	-6.33

^1 ^2005/06 prices. Positive numbers indicate *increased *cost; negative numbers indicate *saving*.

^2 ^Includes cost of non-clinical (training) time

A sensitivity analysis was undertaken to examine how the incremental cost of the service might be influenced by the assumptions made. The impact of variations in the following key cost drivers was considered: the number of hospital admissions avoided; the cost saving associated with each admission; the reduction in length of hospital stay associated with early discharges; the number of nursing home transfers prevented; the length of time for which transfer was avoided; and the level of nursing need (RNCC banding) following transfer to a nursing home. The resulting estimates ranged from a maximum weekly saving of £36.90 per resident to a 'worst case' estimate of £2.70 extra expenditure per resident per week; an overall budgetary impact ranging from £250,000 saved to £18,400 expended per annum. Financial savings are mainly in reduced use of NHS services, although the PCT and LA Adult Social Services both funded the intervention, highlighting the need for partnership working to ensure long-term sustainability.

Balanced against this financial or cost minimisation analysis, there are a number of additional non-cost benefits provided by IRT. As well as improved quality of life, residents benefit from enhanced quality of care with the opportunity for access to a wider range of services. Better preventative and nursing care also enables them to stay in familiar surroundings rather than spending time in hospital or being transferred unnecessarily to a nursing home. For the LA and PCT, the benefits include care staff development, improved job satisfaction, and improved care provision through better partnership working.

## Discussion

Recent NHS policy documents emphasise the need for redesigned health and social care workforces to better meet the needs of older people [19,20]. To date, the residential care home sector has largely been excluded from this discussion. As a result, it has been called the 'twilight zone' in terms of research and policy [21]. Those working in long-term care homes often have limited awareness of national policies and their implications [22]. Furthermore, little is known about the NHS services currently provided to older people in residential homes, or the optimal way in which mainstream NHS services might meet their needs.

In 2000, the Royal College of Physicians, together with the Royal College of Nursing and the British Geriatrics Society, highlighted specific problems with clinical care in residential homes that required resolution [23]. A recent review identifies that a reassessment of the interface between community and residential care is still required to improve access to primary nursing care by older people in care homes [24]. Some of the needs of residents can be met through the care provided by residential home staff themselves, but it is evident that they will also require care from a range of health professionals including district nurses, therapists, GPs, pharmacists and other staff. To date, very few studies have examined the interface of these services with residential care homes. One such study has identified that partnership working between district nurses and residential care home staff largely lacks system and occurs by default [15,25]. This may partly be due to the high demands which community nursing services perceive in residential homes [26,27]. Other research describes poor access to medical services [28]. The impact of care homes (especially nursing homes) on general practitioners' workload has been widely discussed [29-31]. Research from the United States of America (USA) has concluded that organisation of medical input is an important factor influencing nursing home quality [32]. Similarly, in the UK researchers have suggested that medical cover for nursing home residents could be restructured to give improved scope for proactive and preventive interventions [33]. Some researchers have suggested that an allowance be provided to compensate UK GPs for differences in workload associated with care home patients [34]. More recent articles from the USA have discussed whether physician practice might be enhanced by specialising in nursing home care, and by providing payment based on quality-of-care measures [35]. There is more limited research on therapist input to care homes [36,37]. However, enhanced physical and occupational therapy services have been reported to demonstrate a positive effect on functional status and cost of care for long-term residents [38]. At the same time, the cost of occupational therapy is reported to have a negative effect on service use in residential homes [39].

Our estimation demonstrates the likely economic benefits of providing on-site specialist nursing care to residential care homes. The cost of providing the high intensity service (£44.38 per resident per week, 2005/06 prices) should be placed in the context of reported national costs for *ad hoc *community nursing input to local authority residential care homes of between £13.91 and £102.14 per resident per week in 2006/07 [18]. Overall, our findings indicate that in-reach specialist nursing provision for residential homes can be cost-effective. Furthermore, this conclusion is based on activity in the first two years of the new service. As with any service improvement, the efficiency of the intervention is likely to increase over time. There is also the possibility of reduced costs through refinement of team size and membership without a major loss in effectiveness. For example, in the year following the end of the study period (2008) the core IRT nursing team was reconfigured to include a reduced number of nurses, and there was over a 33% increase in annual admissions to IRT.

The role of nurse practitioners in enhancing the clinical care provided to nursing home residents has been explored by other researchers [40]. A number of new models are emerging to enhance the quality of clinical care provided in care homes, although these mainly focus on improving care in nursing homes [21,41-43]. In the USA, interventions such as the geriatric nurse practitioner (NP) for nursing homes are now well established [44-46]. Studies have demonstrated better outcomes for residents with pressure ulcers, incontinence, depression, and aggressive behaviour [47]. Analysis of work patterns indicates that NPs provide a wide range of services including making sick/urgent resident visits, providing preventive care to long-stay residents, hospice care, and wound care [48]. These activities are similar to those reported in the present study. In the US, the EverCare model, which involves case management of frail elderly nursing home residents by nurse practitioners, is reported to have had a positive impact on mortality and preventable hospitalisations [49]. Analysis of EverCare NP work patterns shows that one third (35%) of their working day is spent on direct patient care and the remainder on interacting with nursing home staff, families and physicians [50]. This is similar to the pattern observed in our study. Attempts to transfer the EverCare model to the UK have led to some enthusiastic reports [51] as well as a more cautious assessment [52]. In the US, various strategies for strengthening NP use in nursing homes have been discussed recently and this has led to recommendations about education, acceptable caseloads, and reimbursable visits [53,54].

In a nursing home, NPs can act through empowering nursing staff in the home [55]. For residential care homes, with no in-house nursing staff, a different approach will be required, depending on the nursing needs of residents. Unfortunately, there is limited information on the health needs of residents [56]. The few studies carried out indicate that new admissions to residential care homes can have significant health care needs [57]; and that over half of existing residential home residents have some cognitive impairment [58]. Comparison of preadmission and follow-up health status in a cohort of older people also identifies case-mixes which include higher dependency residents in residential homes and lower dependency residents in nursing homes [59]. This mirrors the finding in the present study that nearly half of residents sampled fell into the mid-RNCC band, indicative of a need for nursing care. Although the current health status of these individuals may not reflect that when they were first admitted, more comprehensive health assessments following admission might enable better nursing care provision. However, to date there have been only a small number of UK studies exploring the use of in-reach nursing teams to improve the care provided in residential care homes [60,61]. The only evaluation of a multidisciplinary care homes support team reports general benefits in London, in particular in managing the interface between nursing homes and primary care [62]. There have been no published studies evaluating such an intervention in residential homes; and none analysing the costs, work patterns and measurable benefits associated with in-reach teams. The present study is the first to report such findings.

As well as improved management of the interface between care homes and primary care, major benefits to residents were identified as the service was established, especially in terms of unnecessary transfers. A number of authors have identified a need to improve transitional care in terms of unnecessary hospitalisations from care homes [63-65]. Much of this research has focused on predicting the risk of admission to hospital [66,67], and of identifying potentially preventable or inappropriate hospitalisations from nursing homes [68,69]. The interface with hospital emergency care is particularly important in this respect [70,71]. Various ways have been considered for reducing hospitalisation rates, including improved clinical pathways [72,73]. There is also some evidence that by providing training for nurses' aides nursing homes may have fewer hospitalisations [74]. Hospitalisations for suspected infections have also been identified as important to control [75-77]. In our study, UTI and chest infections were identified by the in-reach team in a large number of residents.

Previous research studies have highlighted the degree to which hospitalisation affects longer term outcomes in older people [78,79]. It is known that functional decline can occur in a matter of days, emphasising the value of interventions to facilitate timely hospital discharge [80]. Our findings indicate that early discharge to residential care homes can be achieved with the support of an in-reach team. However, a recent Dutch study which assessed a low intensity early discharge model set up in a residential home found that relatively unqualified care home staff and cultural differences between collaborating partners limited the effectiveness of the intervention [81]. Other research on early discharge to nursing homes also concludes that staff such as nurses' aides (who provide the vast majority of direct care to nursing home residents) need training to recognize potential problems after discharge such as the early signs and symptoms of infection [82]. From our study, it is clear that with enhanced training residential homes can perform an important role in terms of post-discharge care; the need for post-acute care for older people has been estimated as up to one-quarter of acute admissions to a UK district general hospital [83].

Finally, it is known that inter-institutional transfers are common in older patients following hospital discharge, with evidence of the need to improve the quality of care in such transitions and ensure patient safety across settings [84]. To date research has focused on admissions to nursing homes from a person's own home [85,86], rather than transfers from residential to nursing home care. Our research demonstrates that such transfers from a residential care home can be safely minimised with structured nursing input.

The main limitation of the present study is that, although activity data were collected prospectively, estimation of benefits such as avoided admissions was necessarily dependent on the retrospective judgement of a review panel. The only way of measuring these benefits prospectively in a robust manner would be through a large scale randomised controlled trial.

## Conclusion

Our research shows that the introduction of a specialist nursing in-reach team for residential homes is likely to be cost neutral and, in all probability, cost saving. As the service has become established, IRT staff costs have reduced and resident case load increased producing an even more favourable financial picture. At the same time, there are additional non-financial benefits provided by such a service. These include the development of new skills in the care home workforce. In the longer term, these might reduce the need for in-reach nursing team input in its present form and produce yet further savings. In addition, enhanced quality of care for residents, with the opportunity for access to a wider range of services, will also result in improved quality of life. In particular, better preventative and nursing care can enable residents to stay in familiar surroundings rather than spending time in hospital or being transferred unnecessarily to a higher dependency setting such as a nursing home. Finally, the observation of residents in the mid-RNCC band indicates that a sustainable solution should also consider placement of individuals into these care homes, including improved training for commissioners and discussion of expectations of long-term residential care with residents and their relatives.

## Competing interests

The authors declare that they have no competing interests.

## Authors' contributions

AS and DW conceived of the study. AS designed the audit assessment, supervised data analysis and drafted the manuscript. SN supervised audit data collection, performed the data analysis, and helped to draft the manuscript. DW participated in the study design and coordination and helped to draft the manuscript. All authors read and approved the final manuscript.

## Pre-publication history

The pre-publication history for this paper can be accessed here:



## Supplementary Material

Additional file 1 Clinical risk stratification – in-reach team (IRT) service. The data provided present the clinical risk tool used in the study and the classification of prevented hospital admissions. Click here for file
