# Supplementary material for: In-reach specialist nursing teams for residential care homes: uptake of services, impact on care provision and cost-effectiveness
Source: BMC Health Serv Res. 2008 Dec 22;8:269. doi: 10.1186/1472-6963-8-269 (PMC2627849; doi:10.1186/1472-6963-8-269)
Supplement: Additional file 1 — Clinical risk stratification – in-reach team (IRT) service. The data provided present the clinical risk tool used in the study and the classification of prevented hospital admissions. [file 1472-6963-8-269-S1.doc]

**Additional file 1: Clinical risk stratification – in-reach team (IRT) service**

**Purpose of Tool: 1. Assess a resident’s risk of Hospitalisation or transfer to a Nursing Home**

**2. Prioritisation for clinical management**

**Resident Name**: ……………………………………………… **Date**:………………………

* ***Please circle all that apply***

| Risk Category | Criteria | Suggested Clinical Strategies for Guidance |
| --- | --- | --- |
| - **HIGH**   **Admit to In-Reach Team (IRT) service if any one or more apply** | - - - 1. Early discharge       2. Significant illness or injury       3. Deterioration of chronic condition       4. Significant change in functional or cognitive  status/decline in condition       5. IV/Subcutaneous therapy       6. Two or more falls in past 30 days       7. Receiving treatment/investigations with significant  risk (e.g. chemotherapy)       8. Significant deterioration in mobility       9. Evidence of significant infection with unexplained  symptoms       10. Terminal Care | - - - - Nursing and physiotherapy assessment       - A minimum of 2 contacts per day. Consider more contacts as needed       - Review medical care plan with GP/DN and revise as indicated. Review changes with  Resident, Family, Care Home Manager and other team members       - Review star chart for trigger factor with Resident, Care Home Manager and NTOW and  key worker to ensure notification of changes       - Review Resident preferences for care with appropriate parties and document changes  including consent/end of life plan       - Review high-risk Residents with GP and all care providers       - Plan care with NTOW and key worker       - Links with CMHCT |
| - **MODERATE**   **If 3 or more apply consider admission to IRT service** | - - - 1. Dehydration       2. Unstable/change in social situation       3. Complex multi-agency involvement       4. History of lung infection in past 60 days       5. Hospital discharge/readmission in past 30 days       6. A & E transfer in past 30 days       7. Change in functional or cognitive status/decline in  condition       8. Minor illness or injury       9. Unexplained Fall or change in mobility       10. Deterioration in condition from baseline status       11. Palliative Care       12. Recurrent infections       13. Weight loss/unexplained and/or continued       14. New Resident to service       15. Significant cognitive disability       16. Unlikely to contact care staff with changes       17. Polypharmacy (6 or more medicines)       18. Undergoing diagnostic studies | - - - - Nursing and physiotherapy assessment       - A minimum of three contact       - Review medical care plan with GP/speciality nurse as needed       - If medical plan of care changes review changes with Resident, family & NTOW       - Review Resident’s preferences and document as needed       - Propose Balance and Agility therapy group       - Complete falls assessment       - Review administration of medication |
| - **LOW** | - - - 1. Needs can be met by other health care providers | - - - - Discuss with and refer to appropriate community team services |

clinical risk stratification /GT/forms/ 19306

**Risk assessed by**: ……………………………. **Agreed with**: ……………………………….. (Title)

Print Name & Title

**Classification: Prevented Hospital Admissions**

The following review classification was used:

**Was hospital admission prevented?**

**Yes**

Where documented evidence clearly indicated that i] the episode of care supported acute illness/injury/palliative care or end of life needs that were clearly discussed with a GP, Community Nurse and/or Registered Care Manager ***and*** ii] there was inter-professional agreement that the resident could only remain in the home with the support of IRT.

**Yes, Probable**

Where documented evidence clearly indicated that i] an episode of care had a focus upon illness prevention and early detection to avoid deterioration of complex ill health, that ii] without intervention of the In-Reach Team ( IRT), was likely to necessitate a hospital admission. Decision-making for these resident episodes was strengthened by the use of the Clinical Risk Stratification (CRS) tool i.e. where there were three or more ‘***moderate risk*’** areas, or a ‘***high risk’*** of hospital admission

**Improbable**

Using the CRS tool, an episode of care that was not complex and rated as ‘***low risk’*** or with two or less ‘***moderate risks’*** with an evidence-based rationale that the resident was unlikely to require an acute hospital admission, even without IRT intervention and admission was not expected or likely. The level of input from IRT was aimed at supporting the residential care staff team to cope with increased needs ranging from reduced nutritional input to reduced mobility. Physiotherapy was often part of this episode. However the team note that in some instances even though admission is not necessary there are negative influences such as the time of day and the day of the week that can result in inappropriate hospital admission. For example the ‘out of hours’ GP services have minimal medical history and residents with high dementia care needs are often difficult to assess. GP’s can be influenced by the residential care home staff to arrange an admission either on a busy Friday evening or over a weekend. This can be due to low staff numbers or shift rotas predominantly made up of ‘agency’. It becomes an easier option to admit the resident to hospital. The existence of the IRT can have a positive influence on these cases.

**No**

Episodes where:

- There was an entirely appropriate hospital admission.
- An admission occurred without the involvement of IRT, e.g. if residential care staff identify an ‘emergency’ situation and report direct to the GP that the resident is ‘unresponsive’ they will be advised to call 999. If an IRT assessment had occurred this may have been managed differently. Use of language has definitely influenced outcomes for residents.
- The illness/injury was not assessed as serious/complex enough and was manageable without hospital admission.
